# Controllable Phase Separation by Boc-Modified Lipophilic Acid as a Multifunctional Extractant

**DOI:** 10.1038/srep17509

**Published:** 2015-12-02

**Authors:** Kai Tao, Lihi Adler-Abramovich, Ehud Gazit

**Affiliations:** 1 Department of Molecular Microbiology and Biotechnology, George S. Wise Faculty of Life Sciences, Tel Aviv University, Tel Aviv 6997801, Israel; 2 Department of Oral Biology, The Goldschleger School of Dental Medicine, Tel Aviv University, Tel Aviv 6997801, Israel; 3 Department of Materials Science and Engineering, Iby and Aladar Fleischman Faculty of Engineering, Tel Aviv University, Tel Aviv 6997801, Israel

## Abstract

While phase separation of immiscible liquid-liquid systems has become increasingly significant in diverse areas, the irreversible nature limits their further application in controllable extraction-concentration or capture-release fields. There is a need for the development of simple, efficient and reversible methods for numerous research and industrial extraction and separation applications. We envisioned Boc-modified lipophilic acids as a simple model for such use based on the studies of the multi-phase transitions of Boc-modified supramolecular polymeric systems. Here, we demonstrate that in the presence of Boc-7-aminoheptanoic acid (Boc-7), phase separation occurs in mixtures of miscible organic solvent and water. The separation behavior was confirmed by differential colorimetric development in aqueous and organic phases using methyl orange staining assays. Component substitution experiments verified that the phase separation results from the subtle balance between the aggregation and the solvation forces of Boc-7, and is reversible by adjusting the solution pH. Owing to the intrinsic hydrophobic properties of the organic phase and the hydrogen bonding-forming ability of the carboxyl group of Boc-7, the phase separation system captures and releases Sudan Red, fluorescein, and streptavidin in a controllable manner. Consequently, a reversible and simple phase separation system can be designed as a multifunctional extractant.

Phase separation formed by two immiscible liquid-liquid phases, such as a chloroform/water system, has been widely used for separation and extraction owing to the intrinsic advantage of using different distribution coefficients for various objectives^1^,^2^,^3^,^4^,^5^,^6^. However, conventional phase separation systems (PSS) are irreversible and not suitable for advanced technologies such as controllable extraction-concentration and capture-release strategies in environmental, medical, and biological applications^4^,^5^. Although ionic liquids could attenuate this disadvantage to some extent, their utilization cannot completely allow for reversiblity^7^,^8^,^9^,^10^. Previously, we demonstrated that Boc-modified diphenylalanine (Boc-FF) can self-assemble into multi-phase supramolecular polymeric assemblies^11^,^12^. Here, Boc-modified lipophilic acids were chosen as minimal models that allow one to study phase behaviors using a simpler system than one based on peptides. In the current work, a pH-dependent reversible PSS, simply composed of hexafluoro-2-propanol (HFIP) and water in the presence of Boc-7, is proposed to controllably capture and release several substances with different properties, as shown in Fig. 1.

## Results and Discussion

Briefly, by pipetting a 50 μL HFIP stock solution of Boc-7 into 950 μL water (1:19, v/v), a PSS was immediately prepared. To investigate the overall process of the phase separation behavior, a pH-dependent methyl orange staining method was employed. As shown in Fig. 2, the supernatant exhibited a transition from red to yellow when pH was set under and above 3.1, respectively, characterizing methyl orange dissolved in an aqueous solution^13^, and indicating that the supernatant was primarily composed of a water phase. While in the other phase, shown as a drop at the bottom of the vial, the color constantly remained red when the pH did not exceed 5.4 (light red at pH 5.4), suggesting that methyl orange was dissolved in a medium other than water. However, when the pH was elevated above 6.1, this shorter phase ceased and the whole solution became homogenously yellow, illustrating that the two separated phases had coalesced into one distinct phase. As a control, a HFIP solution of methyl orange was also shown to turn red when the pH was less than 5.4 and yellow when the pH was elevated above 6.2 (as shown in Fig. S1 in SI). It has been established that the color evolution of methyl orange solution results from an equilibrium shift between the azo moiety and protonated azonium ions^14^,^15^. Since HFIP has a smaller pKa (~9.3) value and is more acidic than water^16^, we speculate that HFIP could promote the equilibrium trend toward protonated azonium ions, inducing the inflection point of color evolution to move to a higher pH value (5.4~6.1) in HFIP than pH ~3.1 in aqueous solution^14^. These results indicate that the separated phase was indeed composed of HFIP. Next, the distribution of Boc-7 was studied using UV-vis absorption. The results indicated that there was no obvious absorption peak between 200 and 300 nm of the PSS supernatant. However, after adjusting the solution to a neutral pH, a remarkably high peak at ~210 nm, characterizing the amide absorption vibrations^17^,^18^, appeared and dominated (as shown in Fig. S2 in SI). This indicates that Boc-7 was primarily distributed in the HFIP phase of PSS, while it was dispersed throughout the entire solution and self-assembled to nanospheres after phase coalescence by pH elevation, similar to traditional surfactants (as shown in scheme 1 and Fig. S3 in SI).

**Figure 1:**
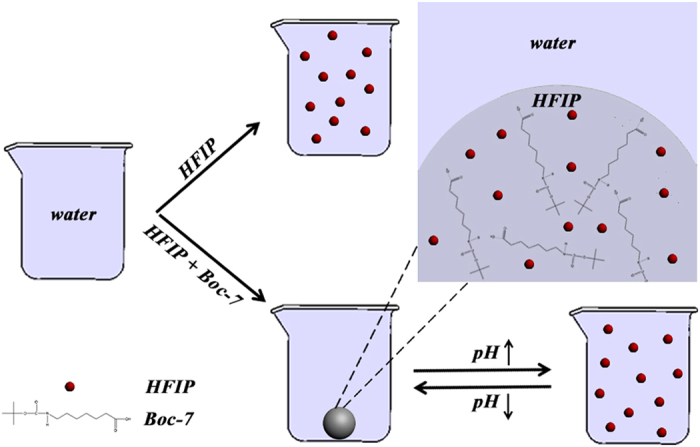
Schematic representation of the pH-dependent reversible PSS. Upper route: HFIP is miscible in water without Boc-7. Lower route: HFIP is separated from water in the presence of Boc-7, which is reversible by adjusting the pH of the solution. The inset shows a magnified, plausible structure in the HFIP phase.

It is well known that HFIP is miscible in water^16^,^19^, as shown in the upper route of Fig. 1. Although Boc-7 exhibits a good solubility in HFIP of more than 100.0 mg mL^−1^, at lower pHs (pH ≤ 5.4) protonated Boc-7 is less hydrophilic and aggregates by hydrophobic interactions. An antagonism exists between the two forces, which, on the one hand inhibited Boc-7 from further aggregation to precipitate, and on the other hand, induced HFIP phase to become hydrophobic. We rationalized that the subtle balance between the two opposing forces resulted in the phase separation, as shown in the lower route of Fig. 1. This balance is stable to some extent. For example, PSS could exist when Boc-7 was replaced with a little less hydrophobic Boc-6 or a little more hydrophobic Boc-8, as shown in Table 1. Nevertheless, the balance is still greatly dependent on the components, including the solution pH, the types of solvent and the protecting groups. When the aggregation propensity of the solute was increased, such as by using poor solvents with high relative polarity (water, TFE, and methanol) to replace HFIP, or by using more hydrophobic Fmoc-7 to replace Boc-7, precipitate or crystals emerged, as shown in Table 1. In contrast, when increasing solvation of the solute, such as using benign solvents with lower relative polarity (DMF, DMSO) to replace HFIP or increasing the volume proportion of HFIP to water to 200/800 (1:4, v/v), homogeneous solutions were obtained and no phase separation was displayed. It should be noted that this force balance is unique and not common, when substituting Boc-7 with other amphiphilic systems, such as amphiphilic peptides (Boc modified diphenylalanine), C-terminal protected amphilhilic amine (methyl 7-aminoheptanoate) or traditional anionic surfactants (sodium dodecyl sulfonate), only precipitates or homogeneous solutions were obtained.

**Figure 2:**
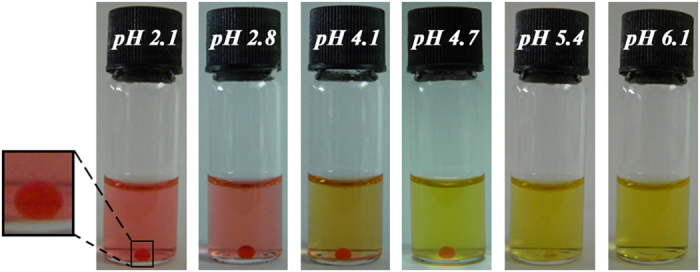
pH-dependent methyl orange staining of the reversible PSS exhibiting a drop at the bottom of the vial when pH less than 5.4, while the phases coalesced into a homogeneous solution at pH 6.1.a The inset shows a magnified view of the separated phase.

Figure 2 shows that pH could also be used to control the force balance. As pH was elevated, the gradually unprotonated carboxyl group of Boc-7 induced the force balance to shift towards the solvation direction, and finally, when pH reached 6.1 or more, the phase separation completely vanished and the system converted to a homogeneous solution. Notably, in this situation the phase transition could be reversed by adjusting pH back to acidic conditions, as shown in the lower route of Fig. 1.

**Figure 3:**
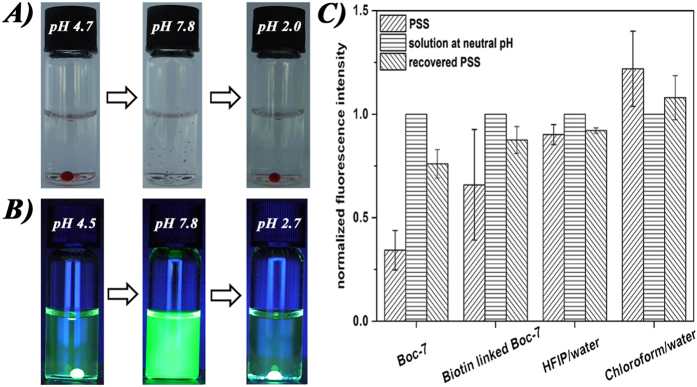
Controllable capture-release of (**A**) Sudan Red, (**B**) fluorescein, and (**C**) streptavidin using the reversible PSS. Note that the sample was viewed under a UV of 312 nm in (**B**), and fluorescence intensities were normalized relative to those of solutions at neutral pH in (**C**).

The reversible feature inspired us to study the applications of this new PSS. Figure 3A shows that after adding Sudan Red stock solution to the PSS, the hydrophobic dye could be captured and confined in the HFIP phase, and by adjusting pH to neutral (pH 7.8) and then to acidic conditions again (pH 2,0), the encapsulated dye could be released and recaptured reversibly. As a control, the dye was always insoluble in HFIP/water without Boc-7 (1:19, v/v) or confined in the chloroform phase in a chloroform/water system (1:19, v/v) regardless of the pH (as shown in Fig. S4 in SI), indicating that Sudan Red was indeed captured by hydrophobic interactions.

In addition to the hydrophobic substances, water-soluble fluorescein could also be extracted and captured, resulting in a visible fluorescence in the HFIP phase and a relatively non-fluorescent aqueous supernatant under UV light, as shown in Fig. 3B. Extraction was also found to be reversible by adjusting the pH to neutral (pH 7.8) and then acidic conditions again (pH 2.7). As a control, fluorescence from blank HFIP/water (1:19, v/v) and chloroform/water (1:19, v/v) always originated from the water phase regardless of the pH (as shown in Fig. S5 in SI), suggesting that the driving force for extraction was not hydrophobic interactions. We hypothesize that hydrogen bonding between carboxyl (Boc-7 and fluorescein) and hydroxyl (fluorescein) groups dominated the driving force, which could be reversibly impaired and restored by controlling the protonation of the carboxyl groups with pH.

Owing to its extraordinarily high affinity to biotin, streptavidin has been widely used in immobilization and functionalization of various active substances^20^,^21^,^22^,^23^. However, because of its ultralow dissociation constant (on the order of ~10^−14^ M), this complex needs very harsh conditions to dissociate, which may easily induce the denaturation of streptavidin^24^. Therefore, a biotin-free substitution, which can capture streptavidin while retaining its activity, is greatly required. Figure 3C shows that, after adding the stock solution of streptavidin, the fluorescence intensity from the PSS supernatant was ~34.2% of that from the solution after adjusting pH to neutral, indicating that ~65.8% streptavidin was captured in the HFIP phase. Note that herein, streptavidin still sustained its activity after release. However, when biotin-linked Boc-7 was used to replace Boc-7 (the synthesis of biotin-linked Boc-7, see the experimental section and Fig. S6 in SI), only 34.1% of streptavidin, approximately half of that when using Boc-7, was captured. We speculate this was because biotin-linked Boc-7 could not form a PSS similar to Boc-7 owing to the distorted force balance after the carboxyl group was blocked. Moreover, after its release, streptavidin lost its activity due to biotin binding. However, when an acidic pH was adjusted once again, the fluorescence intensities from both PSS supernatants (in the presence of Boc-7 and Biotin-linked Boc-7) could not recover to their original values, indicating that the released streptavidin could not be completely re-captured using both systems. Considering the complicated protein conformation, we speculated that the released streptavidin might interact with Boc-7 or HFIP and distort the force balance, leading to the PSS being inferior to its original state. As a control, the fluorescence intensities at acidic and neutral pH from blank HFIP/water (1:19, v/v) or chloroform/water (1:19, v/v) supernatant displayed little difference, indicating that no capture-release of streptavidin occurred in the two systems, as shown in Fig. 3C.

## Conclusions

In conclusion, a Boc-modified lipophilic acid was used as a minimal model to study phase behaviors. Phase separation was performed using miscible HFIP and water in the presence of Boc-7. This phase partitioning resulted from a subtle balance between the aggregation and solvation forces of Boc-7, and could be reversed by easily adjusting the pH of the solution. With its specific features, including the hydrophobicity inside the HFIP phase, the hydrogen bond-forming ability of the carboxyl group of Boc-7, this simple, reversible PSS procedure could be employed as a multifunctional extractant for controllable extraction-concentration and capture-release of substances possessing different properties. Investigations on relevant applications are underway.

## Materials and Experimental Methods

### Materials

Boc-7-aminoheptanoic acid (Boc-7) was purchased from Chem-Impex International Inc., Boc-6-aminohexanoic acid (Boc-6) and Fmoc-7-aminoheptanoic acid (Fmoc-7) were from GL Biochem (Shanghai) Ltd., Boc-8-aminooctanoic acid (Boc-8) was from AnaSpec., fluorescein (free acid) was from Fluka, 1-Biotinyl-3,6-dioxa-8-octaneamine hydrochloride (NH2-PEG2-Biotin•HCl) was from Iris Biotech GmbH., 1-Ethyl-3-(3-dimethylaminopropyl) carbodiimide (EDC) and Streptavidin (DyLight 488 Conjugated) were from Thermo Scientific, and N-Hydroxysuccinimide (NHS) and hexafluoroisopropanol (HFIP) were from Sigma-Aldrich. All chemicals were used as received without further purification, and the water used was processed by a Milli-Q purification system (EMD Millipore, Billerica, Massachusetts, USA) with a minimum resistivity of 18.2 MΩ•cm.

### Reversible PSS Preparation and Characterization

50 μL of a fresh HFIP stock solution of Boc-7 at 100.0 mg mL^−1^ was pipetted into 950 μL water, to prepare a 1 mL solution with Boc-7 at a concentration of 5.0 mg mL^−1^. After having been sealed and stirred vigorously, a PSS was produced. The components of the system were replaced regarding the requirements.

For UV-vis absorption, 200 μL PSS supernatant or solution at neutral pH was pipetted into a 96-well UV-Star UV transparent plate (Greiner BioOne, Frickenhausen, Germany), and the UV-vis absorbance was recorded using a Biotek Synergy HT plate reader (Biotek, Winooski, VT, USA), with a normal reading speed and calibration before reading. Blank HFIP/water mixtures (1:19, v/v) at corresponding pH were used as backgrounds and subtracted.

For atomic force microscopy (AFM), 10 μL of solution at neutral pH was dropped onto a freshly cleaved mica surface and adsorbed for a few seconds. The mica was then rinsed with water and dried gently with nitrogen. A topographic image was recorded under a NanoWizard 3 BioScience AFM (JPK, Berlin, Germany) in the tapping mode at ambient temperature, with 512 × 512 pixel resolution and a scanning speed of 1.0 Hz.

For dynamic light scattering (DLS), a 1 mL solution at neutral pH was introduced into a low-volume disposable sizing cuvette (Sarstedt, North Rhine-Westphalia, Nümbrecht, Germany), and the hydrodynamic diameter was measured using a Zetasizer Nano ZS analyzer (Malvern Instruments, Malvern, UK) at 23.0 °C and a backscatter detector (173°). Three measurements were performed and averaged for accuracy.

For methyl orange staining, 10 μL 0.1 mg mL^−1^ methyl orange aqueous solution was pipetted into the PSS and stirred vigorously. The pH of the supernatant was adjusted to acidic with 1.0 M HCl or neutral with 5.0 M NaOH aqueous solution.

### Controllable Capture-Release Using the PSS

For reversible capture-release of hydrophobic dye, 10 μL 0.1 mg mL^−1^ HFIP solution of Sudan Red was pipetted into the PSS. After having been stirred vigorously, the sample was left standing. The supernatant pH was adjusted to acidic with 1.0 M HCl or neutral with 5.0 M NaOH aqueous solution. HFIP/water with Boc-7 (1:19, v/v) and chloroform/water (1:19, v/v) were used as controls.

For reversible capture-release of fluorescein, 10 μL 0.1 mg mL^−1^ HFIP solution of fluorescein was pipetted into the PSS and stirred vigorously. The supernatant pH was adjusted with 1.0 M HCl or 5.0 M NaOH aqueous solution. The sample was observed under a UV of 312 nm. HFIP/water (1:19, v/v) and chloroform/water (1:19, v/v) were used to replace PSS as controls.

To synthesize biotin-linked Boc-7, 400.0 mg Boc-7 (1.6 mmol), 345.3 mg NHS (3.0 mmol) and 931.4 mg EDC (6.0 mmol) were dissolved in 50 mL water, then sealed and stirred for ester-activation. After reacting for 24 h, the supernatant was removed and the oil-like product was washed with water and vacuum dried. Afterwards, 159.0 mg ester-activated Boc-7 (0.49 mmol) and 200.0 mg NH_2_-PEG_2_-Biotin•HCl (0.49 mmol) were dissolved in sodium acetate buffer (pH 5.0, 6.0 mM), sealed and then stirred for a coupling reaction. After reacting for 72 h, the final product (biotin-linked Boc-7) was extracted from aqueous solution with chloroform and vacuum dried. Product accuracy was confirmed using mass spectroscopy.

For reversible capture-release of streptavidin, 2 μL 1.0 mg mL^−1^ streptavidin stock solution was pipetted into the PSS and stirred vigorously. The supernatant pH was adjusted with 1.0 M HCl or 5.0 M NaOH aqueous solution. Biotin-linked Boc-7 was used to replace Boc-7, or HFIP/water (1:19, v/v) and chloroform/water (1:19, v/v) were used to replace the PSS as controls.

For fluorescence spectroscopy, 600 μL PSS supernatant or solution at neutral pH was pipetted into a 1.0 cm path-length quartz cuvette, and the spectrum was collected on a FL3-11 Spectrofluorometer (Horiba Jobin Yvon, Kyoto, Japan) at ambient temperature. The excitation wavelength was set at 493 nm with a slit of 3 nm, the emission wavelength was set at 495–650 nm with a slit of 3 nm, and data at 516 nm were extracted for comparison. For convenience, the fluorescence intensities were normalized relative to those of solutions at neutral pH. Results were averaged from triplicates for every sample to ensure accuracy.

## Additional Information

**How to cite this article**: Tao, K. *et al*. Controllable Phase Separation by Boc-Modified Lipophilic Acid as a Multifunctional Extractant. *Sci. Rep*. **5**, 17509; doi: 10.1038/srep17509 (2015).

## Supplementary Material

Supplementary Information

## Figures and Tables

**Table 1:** Phase behaviors when replacing Boc-7 with other solutes or HFIP with other solvents.

organic solvent/water (1:19, v/v)	HFIP	water	TFE	methanol	DMSO	DMF
Boc-6	PSS	solution	solution	solution	solution	solution
Boc-7	PSS	precipitate	large crystals	large crystals	solution	solution
Boc-8	PSS	precipitate	precipitate	small crystals	small crystals	small crystals
Fmoc-7	precipitate	precipitate	precipitate	precipitate	precipitate	precipitate

Note that the total solution volume and the concentration of solute are constantly fixed at 1 mL and 5.0 mg mL^−1^, respectively.
