# Supplementary material for: Controllable Phase Separation by Boc-Modified Lipophilic Acid as a Multifunctional Extractant
Source: Sci Rep. 2015 Dec 2;5:17509. doi: 10.1038/srep17509 (PMC4667251; doi:10.1038/srep17509)
Supplement: Supplementary Information [file srep17509-s1.pdf]

Controllable Phase Separation by Boc-Modified Lipophilic Acid as a Multifunctional Extractant

Kai Tao[1], Lihi Adler-Abramovich[1], and Ehud Gazit[*1,2]

[1]Department of Molecular Microbiology and Biotechnology, George S. Wise Faculty of Life Sciences, Tel Aviv University, Tel Aviv 6997801, Israel

[2]Department of Materials Science and Engineering, Iby and Aladar Fleischman Faculty of Engineering, Tel Aviv University, Tel Aviv 6997801, Israel

[*]ehudg@post.tau.ac.il

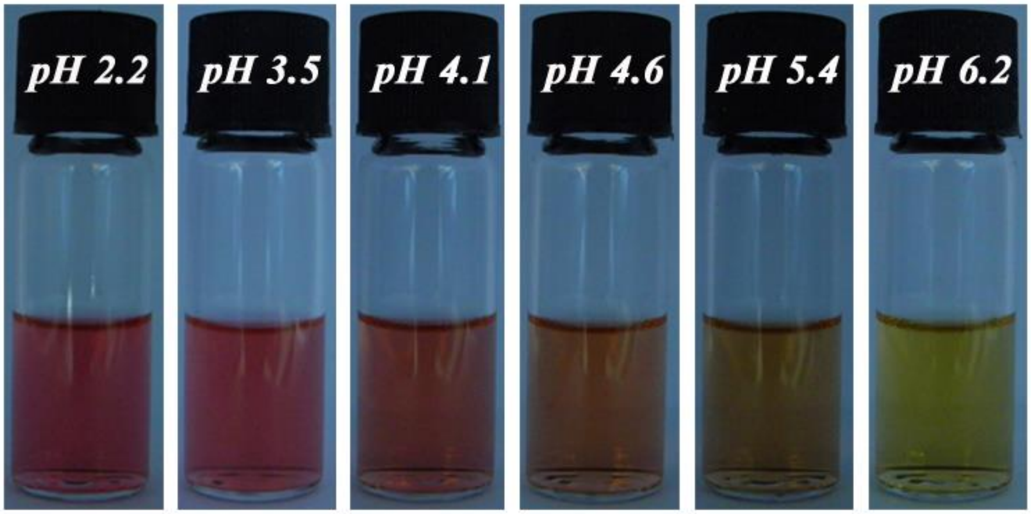

Figure S1. pH-dependent color evolution of a HFIP solution of methyl orange.

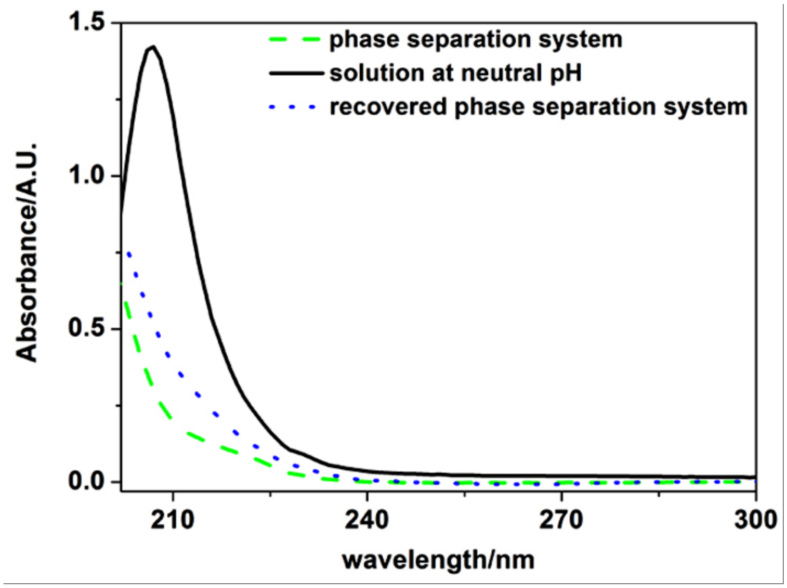

Figure S2. UV-vis absorption of PSS supernatant (green dashed line), solution at neutral pH (black solid line) and recovered PSS supernatant after adjusting pH to acidic conditions again (blue dot line).

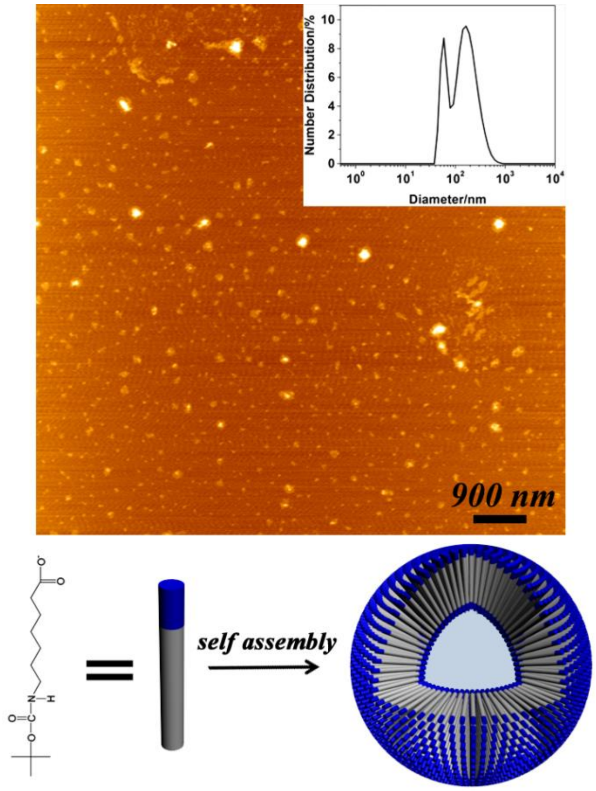

Figure S3. Upper panel: AFM micrograph of nanospheres self-assembled by Boc-7 at pH 7.8. The inset shows the hydrodynamic diameter distribution of the nanospheres measured by DLS. Lower panel: a schematic representation showing the self-assembly mechanism of Boc-7.

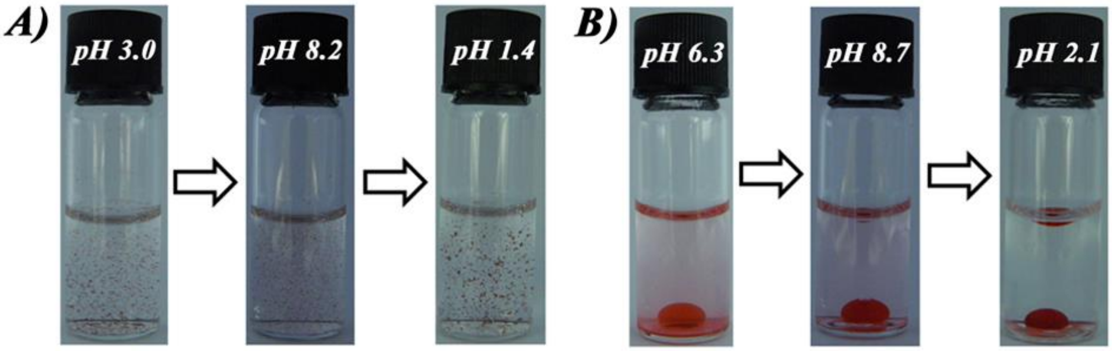

Figure S4. Controls of reversible capture-release of Sudan Red by PSS. (A) Sudan Red in a HFIP/water (1:19, v/v) mixture without Boc-7; (B) Sudan Red in $CHCl_3$/water (1:19, v/v).

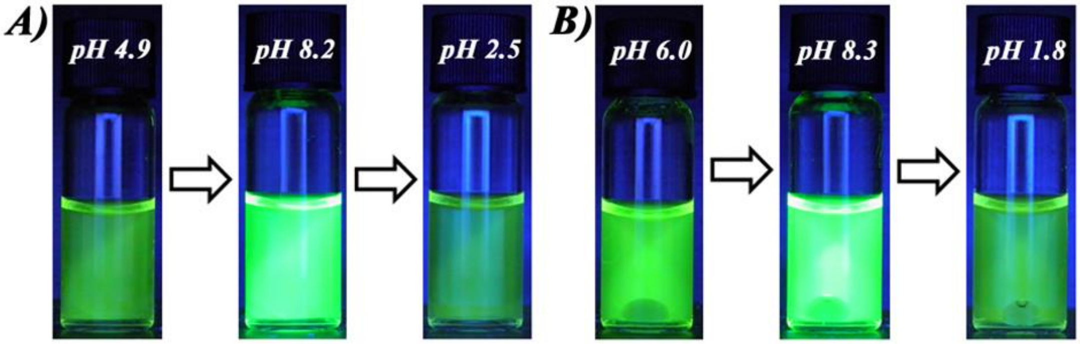

Figure S5. Controls of reversible capture-release of fluorescein by PSS. (A) fluorescein in HFIP/water (1:19, v/v) without Boc-7; (B) fluorescein in $CHCl_3$/water (1:19, v/v). Note that the samples were viewed under UV of 312 nm; the darker, semispherical phase at the vial bottom in (B) is $CHCl_3$.

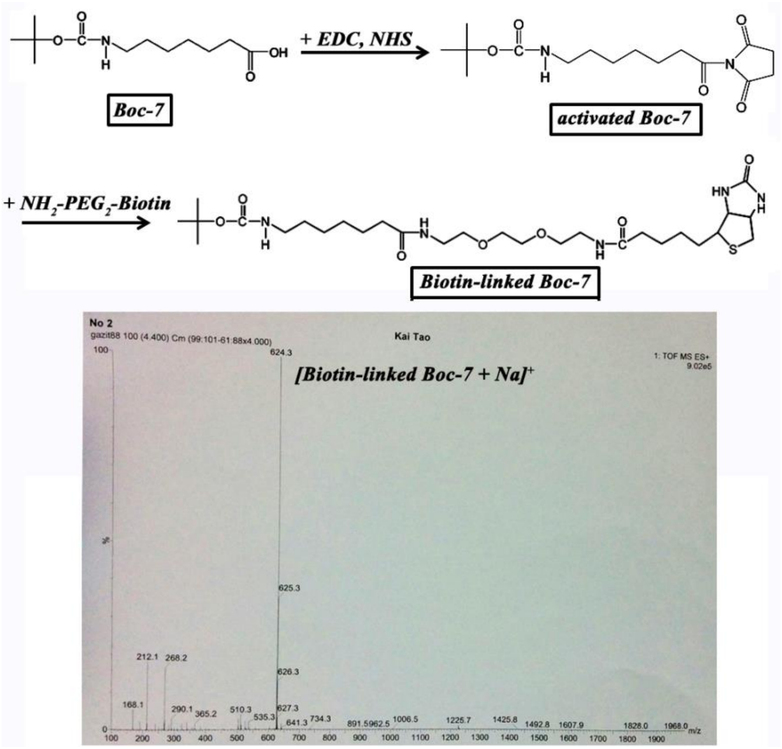

Figure S6. Upper panel: the synthesis routine of biotin-linked Boc-7. First step: activate the carboxyl group of Boc-7 with EDC and NHS to the activated ester. Second step: couple amino-modified Biotin with activated Boc-7 to produce a final product. Lower panel: the MS spectrum of synthesized biotin-linked Boc-7 (MW: 601.80).
